# Transparent by choice: Proactive disclosures increase compliance with digital defaults

**DOI:** 10.3389/fpsyg.2022.981497

**Published:** 2022-10-06

**Authors:** Yavor Paunov, Tobias Vogel, Moritz Ingendahl, Michaela Wänke

**Affiliations:** ^1^Division of Philosophy, School of Architecture and the Building Environment, KTH Royal Institute of Technology, Stockholm, Sweden; ^2^Faculty of Social Sciences, Business Psychology Institute, Darmstadt University of Applied Sciences, Darmstadt, Germany; ^3^Consumer and Economic Psychology, Faculty of Social Sciences, University of Mannheim, Mannheim, Germany

**Keywords:** digital, defaults, nudging, proactive, voluntary, transparency

## Abstract

Default nudges successfully guide choices across multiple domains. Online use cases for defaults range from promoting sustainable purchases to inducing acceptance of behavior tracking scripts, or “cookies.” However, many scholars view defaults as unethical due to the covert ways in which they influence behavior. Hence, opt-outs and other digital decision aids are progressively being regulated in an attempt to make them more transparent. The current practice of transparency boils down to saturating the decision environment with convoluted legal information. This approach might be informed by researchers, who hypothesized that nudges could become less effective once they are clearly laid out: People can retaliate against influence attempts if they are aware of them. A recent line of research has shown that such concerns are unfounded when the default-setters proactively discloses the purpose of the intervention. Yet, it remained unclear whether the effect persists when defaults reflect the current practice of such mandated transparency boils down to the inclusion of information disclosures, containing convoluted legal information. In two empirical studies (*N* = 364), respondents clearly differentiated proactive from mandated transparency. Moreover, they choose the default option significantly more often when the transparency disclosure was voluntary, rather than mandated. Policy implications and future research directions are discussed.

## Introduction

Defaults have been popular online long before nudging was around. Defined as “preselected options chosen by the manufacturer or software developer” (Shah and Kesan, 2006; p. 265), they were initially used to simplify software installation and facilitate product sales. In the wake of the nudging paradigm (Thaler and Sunstein, 2008), defaulting became the most effective technique in the influence toolbox (Hummel and Maedche, 2019; Last et al., 2021). A default nudge typically involves a decision situation where one of the choice options is preselected, but the chooser remains free to select another alternative, i.e., to opt out. Defaults nudges were successfully deployed offline to tackle important issues such as organ donation rates (Johnson and Goldstein, 2003) and carbon emissions (Pichert and Katsikopoulos, 2008). In fact, defaults have been so effective, that using them as compliance boosters has become the norm in US and European policy-making (Jones et al., 2014). Their offline popularity quickly spread back online to the emerging field of digital nudging. Online default nudges share the same goal with their offline counterparts–to guide people toward self-beneficial choices by harnessing “the power of inertia” (Thaler and Sunstein, 2008, p. 8). The use cases for digital default nudges are numerous, ranging from increasing carbon offset contributions (Franzoi and vom Brocke, 2022) to enhancing user privacy (Baek et al., 2014). In general, digital defaults are effective (Hummel and Maedche, 2019), and their popularity as an online influence tool increases.

Despite their widespread adoption, both digital and conventional defaults face considerable criticism on ethical grounds. Since the target of an offline default is largely unaware of the influence attempt and the psychological processes behind it (Hansen and Jespersen, 2013), some researchers have argued that defaults limit people's ability to exercise free choice (Smith et al., 2013). Hence, offline default nudges are often described as paternalistic and restrictive (Bruns and Perino, 2019). Accordingly, survey data show that defaults are viewed less favorably and perceived as more autonomy threatening than other influence aids (Jung and Mellers, 2016).

Criticism toward digital defaults is less about lack of awareness, and more about the way they are implemented in the decision environment. Most digital defaults are presented as “recommended choices,” and the pre-selections are clearly visible checkboxes or radio buttons. What is less visible, however, is how the set of choice options is derived. Since the online environment can be quite complex (Wan et al., 2007), decision making is often assisted by algorithmic recommendation systems (Punj, 2012). These systems derive a personalized set of alternatives for the users, and often preselect one to nudge the users to choose it (Bothos et al., 2016; Bauer and Schedl, 2017). However, even if the algorithm pre-selected the most beneficial option for the user, the logic behind the pre-selection is rarely disclosed. Moreover, the best choice for the individual can be entirely missing from the derived set of alternatives if the algorithm's personalization logic is flawed or inapplicable to a group of users (Jesse and Jannach, 2021). Therefore, despite being aware of the default, the users are still not in complete control of their decision.

An intuitive solution would be to make the implementation of offline and digital defaults more transparent. In fact, many researchers have already called for an increase in transparency when nudging with offline defaults, especially on the governmental level (Sunstein, 2015; Ivanković and Engelen, 2019). Online, the need for transparency has already been recognized, and official directives mandate transparency when defaults are used, especially in the fields of data privacy and information sharing (EU Electronic Privacy Regulation EPR, 2017). In the common case, regulated transparency is implemented by providing the users with access to lengthy legal documentation (Alemanno and Spina, 2014), or to relevant open data (Greveler, 2017).

However, some researchers have also expressed concerns that transparency might harm the effectiveness of default nudges. Krijnen et al. (2017) speculated that people may actively opt-out from a pre-selection once the influence attempt is disclosed if existing strong attitudes against the promoted decision outcome are invigorated. Bovens (2009) also theorized that once a default is made transparent, people will realize they were not fully in control of their actions and tend to self-correct their agency. Hence, he argued that non-transparent nudges, such as defaults, work “better in the dark” and should become increasingly ineffective as transparency is introduced. Reactance theory (Brehm, 1966) would make even stronger predictions, linking resistance to the mere presence of an influence attempt.

The current empirical evidence does not back such theorizing. Several experimental studies report mostly null findings, and find no evidence for a negative effect of transparency on offline and digital default effectiveness (Loewenstein et al., 2015; Steffel et al., 2016; Bruns et al., 2018). A recent line of research (Paunov et al., 2019a,b, 2020) even showed that when policymakers proactively disclosed the purpose of the intervention, the effectiveness of the default increased. In multiple studies, the authors asked their participants to commit time to participate in an experiment and preselected one of the choice options. Disclosing why the option was defaulted almost tripled compliance compared to a conventional default. The sizable compliance boost was partially explained by people's perceptions about the policy endorser. When the reason for implementing the default was disclosed proactively, people perceived the default-setter as more sincere and responded with increased compliance.

In all studies cited so far, the experimenters compiled various transparency disclosures and presented them before (Hokamp and Weimann, 2022), after (Loewenstein et al., 2015), or simultaneously with (Wachner et al., 2020) the default nudge. Preferences for the default option were then compared with choices in a conventional, non-transparent default condition. While these are valid designs to isolate transparency effects, they do not reflect the current practice of transparent policy-making. As mentioned before, the norm in most democratic systems is to provide constituents with access to policy-relevant information. In Europe, this norm is formalized and implemented in legislation as a fundamental citizen right of information access [EU Treaty, 2016, Article 1(2); Article 15(3)]. In the digital domain, all public and private agents are subject to strict data protection regulations (GDPR, 2016), and compliance is strictly enforced.

It is surprising then, that previous research on transparent defaulting has not emulated situations in which the mandated nature of transparency is made apparent. Would users differentiate between voluntary and mandated transparency disclosures while being nudged with defaults online? If so, would compliance differ depending on the type of disclosure, and how? This is a relevant line of inquiry, since research on reciprocity (Gouldner, 1960) and two-sided persuasion (for a comprehensive review see Eisend, 2006) shows that people respond positively to relevant disclosures, but only if the information is disclosed voluntarily (Jones and Davis, 1965; Schnackenberg and Tomlinson, 2016). Indeed, evidence from consumer research shows that forced (or mandated) disclosures have little effect on behavior (John et al., 2016; Mohan et al., 2019).

In the following research, we explore the effects of mandated and voluntary transparency disclosures on compliance with digital default nudges. In two studies, we implement online defaults aimed at encouraging student research participation. We assume that when users are nudged with an online default, they will: (H1a) differentiate between obligatory (mandated) and voluntary (proactive) transparency disclosures, and (H1b) will comply more, when the accompanying transparency message is shared proactively, rather than by obligation. We chose the academic backdrop for two main reasons: First, it is a common occurrence that students need mandatory ECTS credits, which can be obtained only by participating in online research. However, the task of choosing among the numerous available surveys can be quite overwhelming, when combined with the pressure to participate. Hence, a default might facilitate the process. Second, online research participation is on a global decline (Arfken and Balon, 2011), and the domain can benefit from effective decision aids.

## Method

### Design and participants

We tested our hypotheses in two online experimental studies (*N* = 367), where we compared the effects of proactive and mandated transparency disclosures on choosing a defaulted option. The two studies followed a very similar procedure and are thus reported together. Both had the same three-group between-participants design, where a conventional (non-transparent) default condition was contrasted with a mandated (obligaroty) transparency condition, and a proactive (voluntary) transparency condition.

The two studies were conducted as a part of a larger research project, which determined the final sample size. For each experiment, English speakers were recruited *via* Prolific Academic to participate in a study on human perception and decision processes for a payout of ~1.41£ for a duration of ~10 min. A sensitivity analysis in G^*^Power (Faul et al., 2007) revealed that our smallest sample (N = 163) was sufficient to detect small to medium effect sizes (w = 0.24) in a standard contingency table Chi-Square test with 3 x 2 cells (condition x default chosen). For Study 1, we recruited 198 participants (126 female, 68 male, 4 diverse), MAge = 31.57, SDAge = 10.93, 192 native speakers. For Study 2, we recruited 163 respondents, (75 female, 87 male, 1 diverse), MAge = 33.19, SDAge = 12.98, 158 native speakers.

### Procedure

We adapted the experimental procedure from previous research on the effects of transparency disclosures in digital settings (Paunov et al., 2020). After signing a standard informed consent, the users were directed to an online research participation platform and asked to choose a study to complete. The studies were organized in categories, ordered by duration (<9 min, 9–11 min, 11–13 min, 13–15 min, >15 min). The participants were informed that they would receive the agreed payment for 11 min, no matter if they completed a longer or a shorter study. Across conditions, the 11–13 min category was preselected (for a visual, see OSF directory in Data Availabity Statement).

**Figure 1:**
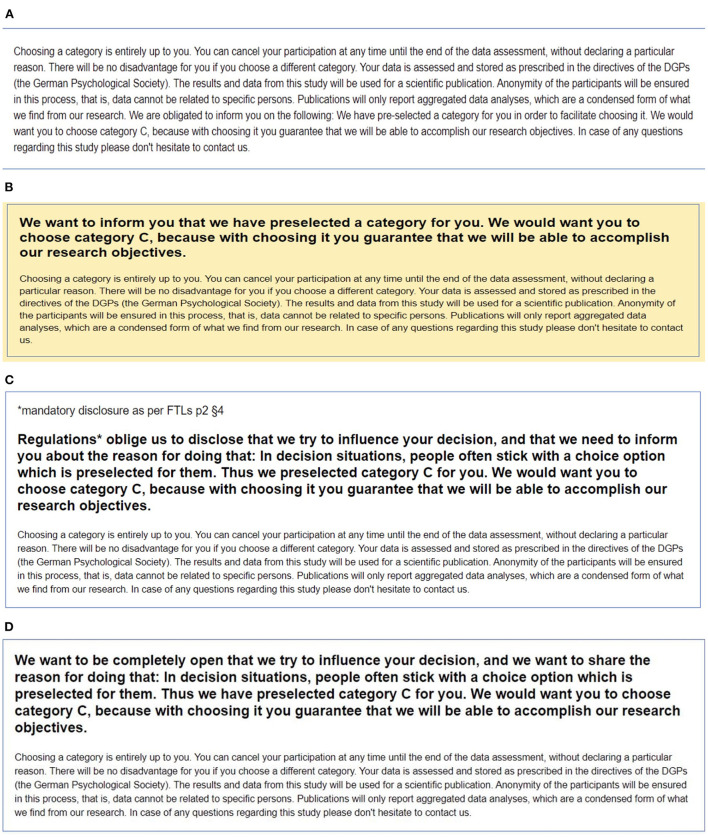
Screenshots from the information disclosures in transparency conditions per experimental study. **(A)** Mandatory transparency condition Study 1. **(B)** Proactive transparency condition Study 1. **(C)** Mandatory transparency condition Study 2. **(D)** Proactive transparency condition Study 2.

In all conditions, the default was accompanied by a text message informing about the relevant data use and storage regulations. In the mandated transparency condition, the text also explained the purpose of the default intervention. Importantly, the message explicitly stated that the disclosure was made due to legal obligation. In the proactive transparency condition, an identical text was presented, this time explicitly claiming that the disclosure was included voluntarily by the default-setter. The respective transparency disclosures are presented in Figure 1 and in the OSF directory (see Data Availability Statement).

The salience of the proactive transparency message was varied between studies to test if the voluntary aspect of the disclosure needs to be additionally emphasized to produce compliance. In Study 1, the proactive disclosure was written in large font and framed in a vibrant color. In Study 2, both disclosures were made equally salient, as important parts of the text were written in bold and in large font. After choosing a task category, all respondents answered a set of questions to verify whether they had acknowledged the default, understood the content of the respective transparency information, and identified the type of transparency (proactive or mandated) correctly.

## Results and Discussion

### Main analysis

In both studies, exact binomial tests were used to establish the presence of a main default effect. In both Study 1 (71%) and Study 2 (56%), participants selected the defaulted option above chance levels (20%), with both *p* < 0.001. Hence, we report a significant main effect of defaulting in both studies, and we can move on to testing whether choices were affected by the respective transparency disclosures.

Table 1 shows that in both studies choices for the default option were highest in the voluntary transparency conditions. In Study 1, preferences for the default under voluntary transparency (94.3%) were more approximately 30% higher than in the mandatory transparency condition. In Study 2, the trend persisted, with roughly 20% more participants choosing the default when it was disclosed proactively, rather than by obligation.

**Table 1:** Choices for the default option by experimental condition.

	**Condition**		
**Study**	**Conventional default**	**Mandatory transparency**	**Proactive transparency**
Study 1	60.7% (37/61)^*^	56.7% (38/67)	94.3% (66/70)
Study 2	44.4 % (24/54)	52.5% (31/59)	71.4 % (40/56)

* Default choices per condition to total N per condition in parentheses.

Two separate Pearson's Chi-squared tests revealed that these differences were statistically significant in Study 1[χ2(2) = 28.36, *p* < 0.001], and in Study 2 [χ2(2) = 8.63, *p* = 0.013]. In order to test the specific differences between conditions, we ran binomial logistic regressions with the binary decision (1 = choosing the default option; 0 = selecting an alternative) as a criterion. In both studies, the criterion was predicted from two Helmert contrasts. The first contrast accounted for differences between the conventional default condition and both transparent conditions (proactive and mandatory transparency = 1, conventional default = −2), thus indicating how transparency in general affects choices for the default. The second contrast accounted for differences between the two transparent groups (proactive = 1, mandatory= −1, conventional = 0). It indicates whether a given type of transparency leads to more or less choices for the default option.

The analysis yielded the following effects. The first contrast was significant in both Study 1 (b = 0.74, SE = 0.26, z = 2.85, *p* = 0.004) and Study 2 (b = 0.24, SE = 0.11, z = 2.17, *p* = 0.030). Hence, the participants in the transparent conditions were significantly more likely to stick with the default option than those in the conventional default conditions.

Critically for our research objectives, the second contrast was also significant in both Study 1 (b = 2.37, SE = 0.58, z = 4.10, *p* < 0.001) and Study 2 (b = 0.41, SE = 0.20, z = 2.07, *p* = 0.039). Hence, in both studies people chose to stay with the defaulted option significantly more often when the transparency information was disclosed proactively, rather than by obligation, in support of Hypothesis 1b.

### Attention checks

After the choice task, we checked whether the respondents had acknowledged the pre-selection in the decision environment. They had to select one of four statements, where the correct one stated that a choice option had been preselected. 87.88% of the participants in Study 1 and 84.61% of the participants in Study 2 chose the correct statement and passed the check. The success rate did not differ between conditions, χ2(2) = 2.52, *p* = 0.284 (Study1), and χ2(2) = 1.97, *p* = 0.373 (Study 2). Hence, the respondents were aware of the default across conditions and studies.

The participants also had to identify the purpose of the default by selecting it from a set of four options. Note that this purpose had only been disclosed in the two transparency conditions. In Study 1, the majority of respondents in the voluntary transparency condition identified the purpose of the default correctly (88.57%). Correct responses in the mandatory (41.79%) and the conventional default conditions (32.79%) where less common, χ2(2) = 48.85, *p* < 0.001. This can be attributed to the difference in salience between the two transparency disclosures in Study 1. When the salience level was unified for both disclosures in Study 2, the correct responses in the voluntary (83.93%) and the mandatory transparency conditions (79.66%) became equal, and were still significantly higher than in the conventional default condition (50.00%), χ2(2) = 18.46, *p* < 0.001.

Last, we assessed the participants' understanding of the additional text information (data security regulation, data anonymity, etc.), which was presented in all experimental conditions. No significant differences were detected between conditions across both studies (all *p*'s > 0.934), indicating that the respondents have processed the additional information equally well.

### Manipulation checks

After completing the attention check, the participants expressed their agreement with a set of 4 additional statements. Two statements captured the respondents' global assessment of the extent to which the purpose of the default had been made transparent [e.g., “It was unclear to me why option C was preselected” (reversed)]. Higher scores on this scale indicated higher agreement that the default was implemented in a transparent way. Two further statements captured the respondents' opinion on whether the endorser had disclosed the purpose of the intervention proactively (e.g., “The policy endorser wanted me to know why category C had been pre-selected”). Higher scores on this scale meant stronger belief in the voluntarity of the disclosures. In Study 2, we added an additional item to each scale. In both studies, the internal consistency for the two scales was sufficient (Cronbach's alpha >0.72). The full text of the items in each scale is provided in the OSF registry.

In both studies, we separately regressed the participants' scores on the same Helmert contrasts, used in the main analysis. The respective estimates, marginal means, and standard errors are provided in the OSF registry. Across studies, the respondents from the transparency conditions believed that the default had been more transparent, compared to the respondents from the conventional default conditions. Both in Study 1 (t = 4.27, *p* < 0.001) and Study 2 (t = 7.46, *p* < 0.001), these differences were statistically significant. In Study 1 (t = 3.68, *p* < 0.001), but not in Study 2 (t = 0.62, *p* = 0.538), the difference was also significant between the two transparency conditions. Crucially for our voluntarity manipulation, the participants in the proactive transparency conditions agreed significantly more that the default disclosure was voluntary, compared to those in the mandatory transparency conditions. This was true in both Study 1 (t = 6.60, *p* < 0.001) and Study 2 (t = 2.23, *p* = 0.027).

In summary, the results indicate that our transparency manipulation was successful. In both transparency conditions the respondents believed that the endorser made the purpose of the default sufficiently transparent. Importantly, participants in the voluntary transparency condition were more likely to think that the disclosure was made proactively, rather than by obligation, thus supporting Hypothesis 1a.

## General discussion

In two experimental studies (*N* = 364) we deployed a digital default nudge in the context of online research participation. Across experiments, we demonstrated that making digital defaults transparent is a viable way of increasing respondent compliance. Pertinent to our research objectives, we showed that people clearly differentiate between proactive and obligatory transparency disclosures. They complied significantly more, when the endorser shared the purpose of the intervention voluntarily. In a field, where transparency is in high demand (Ivanković and Engelen, 2019), and mandated information disclosures are not uncommon (Loewenstein et al., 2013), we believe that our findings make a meaningful contribution to the literature.

Limited exploratory evidence also indicated that increased visual salience can help people perceive the respective disclosures as clearer and more transparent. This is partially reflected in the compliance differences between the transparency conditions in Study 1. However, the increase in compliance caused by the proactive transparency disclosure remained impressive even when the visual salience was unified between conditions (Study 2).

While the results advocate a proactive approach to transparency, there are some limitations to their generalizability. First, compliance with transparent defaults could strongly depend on the purpose of pre-selection. If that purpose is at odds with the respondents' self-interests (Steffel et al., 2016) or deep-rooted convictions (Krijnen et al., 2017), transparency might lose its effectiveness altogether, independently of the voluntarity of the disclosure. We aimed to keep our default manipulation within the ethical frame of the nudging paradigm, i.e., to direct choices toward a goal, which people already consider beneficial (Thaler and Sunstein, 2008). However, we acknowledge that this is unlikely to be the case for commercial or political defaults.

Second, we acknowledge that the effect might depend on the degree, to which the respondents think that the voluntarity of the disclosures is genuine. It is likely that such judgments are context-dependent. A large number of defaults are implemented online to facilitate the acceptance of behavioral tracking scripts, or “cookies.” They often contain messages like “We care about your privacy” to give the impression that the site owners disclose the respective privacy information voluntarily. As mentioned previously, such “disclosures” are necessitated by law, and failure to present them can be extremely costly [Art. 84(3) GDPR, 2016]. Though differences between countries exist, the average European citizen is aware of GDPR (Rughiniş et al., 2021). Hence it is unlikely that people would believe that disclosures in the data privacy domain are made out the endorsers' good will.

Third, we note that the respondents' beliefs about the sincerity of a given (voluntary) disclosure may also depend on the setting, in which the default is implemented. We chose an academic backdrop in an attempt to increase student participation in online research. While doing so can be beneficial for both students and researchers, we understand that people generally trust academics more than commercial or political actors (Pew Research Center, 2022), and could be thus inclined to consider their disclosures more genuine. One would imagine that people will be more skeptical toward disclosures coming from commercial digital agents, even if the agents proactively disclose their intentions when defaulting. Hence, future research should explore possible interactions between default setting and perceptions of disclosure voluntarity on compliance.

Despite the abovementioned limitations, we think that our findings stand for the inclusion of proactive, voluntary transparency disclosures in digital defaults. At the least, we show that policy makers should not shun away from proactively sharing their intentions when nudging in the academic domain. The effort to make the intervention transparent by choice will be recognized by the nudged, and can be rewarded with increased compliance.

## Data availability statement

The datasets presented in this study can be found in online repositories. The names of the repository/repositories and accession number(s) can be found at: https://osf.io/ntbgq/?view_only=095ff2d276634e009cc07279a8069808.

## Ethics statement

Ethical review and approval was not required for the study on human participants in accordance with the local legislation and institutional requirements. The patients/participants provided their written informed consent to participate in this study.

## Author contributions

YP was partially responsible for creating the experimental paradigm and editing the experimental materials. He made the main contribution toward writing the paper. TV was partially responsible for conceptualizing the experimental ideas and creating the experimental materials. MI was partially responsible for creating the experimental materials, as well as for the data analysis and writeup. He contributed toward writing the analysis section of the paper. MW was responsible for conceptualizing the main research goals and participated in the manuscript writing. All authors contributed to the article and approved the submitted version.

## Conflict of interest

The authors declare that the research was conducted in the absence of any commercial or financial relationships that could be construed as a potential conflict of interest.

## Publisher's note

All claims expressed in this article are solely those of the authors and do not necessarily represent those of their affiliated organizations, or those of the publisher, the editors and the reviewers. Any product that may be evaluated in this article, or claim that may be made by its manufacturer, is not guaranteed or endorsed by the publisher.
